# Salivary cortisol response to infant distress in pregnant women with depressive symptoms

**DOI:** 10.1007/s00737-014-0473-0

**Published:** 2014-10-29

**Authors:** Susannah E. Murphy, Elizabeth C. Braithwaite, Isabelle Hubbard, Kate V. Williams, Elizabeth Tindall, Emily A. Holmes, Paul G. Ramchandani

**Affiliations:** 1 Department of Psychiatry, University of Oxford, Oxford, United Kingdom; 2 Department of Psychology, University of Bath, North East Somerset, United Kingdom; 3 MRC Cognition and Brain Sciences Unit, University of Cambridge, Cambridge, United Kingdom; 4 Academic Unit of Child and Adolescent Psychiatry, Imperial College London, London, United Kingdom

**Keywords:** Pregnancy, Cortisol, HPA axis, Depression, Anxiety, Stress

## Abstract

The Hypothalamic-Pituitary-Adrenal (HPA) axis has been proposed as a potential underlying biological mechanism linking prenatal depression with adverse offspring outcomes. However, it is unknown whether the reactivity of this system to stress is altered in pregnant women experiencing depression. The objective of this study was to investigate whether salivary cortisol response to a distressed infant film is enhanced in pregnant women with symptoms of depression compared with non-depressed controls. Salivary cortisol and subjective mood responses to the film were measured in 53 primiparous women, between 11 and 18 weeks gestation. Both groups showed similar increases in state anxiety in response to the film, but there was a significantly increased cortisol response in women experiencing symptoms of depression. Depression during pregnancy is associated with increased reactivity of the HPA axis. This is consistent with altered HPA axis functioning being a key mechanism by which prenatal mood disturbance can impact upon fetal development.

## Introduction

Maternal depression during pregnancy is associated with increased risk of poor birth outcomes (Lobel 1994), infant behavioural, cognitive and emotional problems (O’Connor et al. 2002; Talge et al. 2007) and later diagnosis of psychiatric disease in adulthood (Van den Bergh et al. 2007). However, the underlying mechanisms by which prenatal depression may directly influence fetal development are not well understood. The Hypothalamic-Pituitary-Adrenal (HPA) axis of both the mother and child has been proposed as a potential underlying biological mechanism linking prenatal depressed mood with adverse offspring outcomes. During pregnancy, there is transplacental transfer of glucocorticoids, including cortisol, and this plays an important role in normal fetal organ maturation and preparing the fetus for postnatal life (Amiel-Tison and Pettigrew 1991). However, excessive levels of these hormones may have negative influences on fetal development, and there is evidence to suggest altered glucocorticoid exposure during gestation can permanently alter HPA function and lead to chronically elevated levels of glucocorticoid activity postnatally (Challis et al. 2001). Thus, a theoretical model proposed to explain the association between maternal prenatal mood and infant outcomes suggests that maternal prenatal depression results in excessive levels of maternal cortisol, which leads to ‘programming’ of the offspring HPA axis during fetal development (Glover et al. 2010).

Although cortisol is known to be a key mediator of the stress response in humans, there are dramatic functional changes in this system across the course of pregnancy, with a 2–4-fold increase in maternal cortisol by full term. It is therefore important to establish the extent to which maternal stress during pregnancy is associated with altered levels of cortisol. Studies investigating the association between self-report measures of maternal stress (such as life events, perceived stress and daily hassles) and basal levels of maternal cortisol have yielded mixed results, with studies reporting no association (Petraglia et al. 2001) and some reporting a small association between high levels of stress and increased levels of maternal cortisol (Buitelaar et al. 2003; Diego et al. 2006). However, studies looking at alterations in diurnal patterns of cortisol levels have been more consistent, with reported associations between a flatter diurnal decline in cortisol levels and trait anxiety (Kivlighan et al. 2008), stressful life events (Obel et al. 2005) and depression (O’Connor et al. 2014; although see Suglia et al. 2010). Consistent with this, O’Keane et al. (2011) reported increased evening cortisol in depressed pregnant women compared with non-depressed controls. Lower morning cortisol levels have also been associated with depression (O’Connor et al. 2014; Shea et al. 2007) and cumulative stress (Suglia et al. 2010) in high-stress populations.

Less is known about how HPA axis reactivity in response to acute stress changes in pregnancy, and whether this is also affected by mood disturbance. Consistent with the increase in basal levels of cortisol that occurs during pregnancy, there is some evidence to suggest that the HPA axis becomes less reactive to acute psychosocial stressors in the later stages of pregnancy (Kammerer et al. 2002). There is also some evidence that cardiovascular responses to acute stress also attenuate as pregnancy progresses (Entringer et al. 2010; Matthews and Rodin 1992). It has been suggested that a dampening of stress responsivity during pregnancy, may serve a protective function, as over exposure to maternal stress hormones and alterations in cardiovascular responses, including uterine blood flow, may disrupt normal fetal development (Christian 2012). However, others have reported robust cortisol responses to acute laboratory stressors that persist even in the late stages of pregnancy (De Weerth et al. 2007; Nierop et al. 2006). Interestingly, even in studies where there is an overall reduction in stress reactivity at a group level, there are individual participants for whom the stress response remains strong (Kammerer et al. 2002). One possibility for this variation in findings in the field is that there are other factors, such as maternal mood, that influence stress reactivity in pregnancy that have not been controlled for in previous studies.

A limited number of studies have investigated mood-related changes in cardiovascular reactivity to acute stress during pregnancy (Monk et al. 2000; Pearson et al. 2012). The largest study, to date, suggested anhedonic symptoms of depression during pregnancy are associated with increased systolic blood pressure in response to infant distress stimuli (Pearson et al. 2012). However, a critical gap in the literature is that it is currently unknown how maternal HPA axis reactivity in response to stress is altered in women who experience depression during pregnancy. In the current study, salivary cortisol response to a film of distressed (crying) infants was examined in two groups of pregnant women with and without depressive symptoms. The distressed infant film was chosen as an acute stress induction because there is evidence that infant-related stimuli may be a particularly potent probe of mood-related differences in responsivity during pregnancy (Pearson et al. 2012). It was predicted that cortisol responses would be increased in women who scored higher on a measure of depressive symptoms. Given evidence that HPA axis activity differs by parity status (Rasheed 1993; Vleugels et al. 1986), only women pregnant with their first baby were included in the study.

## Methods

### Participants

Fifty-four women were recruited during routine early pregnancy ultrasound visits, through the Obstetric Ultrasound Unit of the John Radcliffe Hospital in Oxford, UK. Inclusion criteria were as follows: 11–18 weeks gestation, primiparity and sufficient fluency in English to understand task instructions. Women were on average at 14.9 weeks of gestation (SD 10 days; range 11.7–17.6 weeks) at the time of testing and their mean age was 31 years (SD 4 years; range 20–40 years). All had single fetal pregnancies. See Table 1 for further participant characteristics. The study was approved by the Research Ethics Committee Oxford B. All participants gave written informed consent and were reimbursed for their time. One woman was not able to provide sufficient saliva to allow cortisol assay and so was excluded from the analysis, leaving a total of 53 participants.

**Table 1:** Participant demographics

	Total sample (*n* = 53)	Depressive symptom group (*n* = 14)	Non-symptom group (*n* = 39)
Age, years (range)	30.9 (20–40)	30.6 (21–40)	31.0 (20–37)
Gestation, days (range)	104.9 (84–123)	101.3 (88–116)	106.1 (84–123)
Spielberger Trait Anxiety Inventory, mean (range)	35.7 (20.5–59)	42.4 (30–59)	33.3 (20–53)
Edinburgh Postnatal Depression Scale, mean (range)	7 (0–17)	13.4 (10–17)	4.7 (0–9)
Marital status, *n* (%)			
Married	38 (71.7)	9 (64.3)	29 (74.4)
Cohabiting	13 (24.5)	3 (21.4)	10 (25.6)
Stable relationship not cohabiting	2 (3.8)	2 (14.3)	0
Single	0	0	0
Employment, *n* (%)			
Full time	46 (86.8)	12 (85.7)	34 (87.2)
Part time	5 (9.4)	2 (14.3)	3 (7.7)
Unemployed	0	0	0
Student	0	0	0
Self-employed	2 (3.8)	0	2 (5.1)
Education (highest qualification), *n* (%)			
GCSE	4 (7.5)	1 (7.1)	3 (7.7)
A-level	3 (5.7)	0	3 (7.7)
NVQ	3 (5.7)	2 (14.3)	1 (2.6)
Undergraduate degree	16 (30.2)	6 (42.9)	10 (25.6)
Postgraduate degree	25 (47.2)	5 (35.7)	20 (51.3)
Other	2 (3.8)	0	2 (5.1)
Ethnicity, *n* (%)			
Caucasian	51 (96.2)	13 (92.9)	38 (97.5)
Other	2 (3.8)	1 (7.1)	1 (2.6)
Unplanned pregnancy, *n* (%)	5 (9.4)	2 (14.3)	3 (7.7)
History of mood disorder, *n* (%)	10 (18.9)	8 (57.1)	2 (5.1)
Smoker pre-pregnancy, *n* (%)	7 (13.2)	3 (21.4)	4 (11.3)
Smoker during pregnancy, *n* (%)	1 (1.9)	0	1 (2.6)
Alcohol consumption during pregnancy *n* (%)			
None	44 (83)	12 (85.7)	32 (82.1)
Less than 2 units per week	5 (9.5)	2 (14.3)	3 (7.7)
More than 2 units per week	4 (7.6)	0	4 (10.3)

*GCSE* general certificate of secondary education, *A level* advanced level, *NVQ* national vocational qualification

### Procedure

Women who met the inclusion criteria and expressed an interest in taking part in the study were contacted by a researcher and invited to attend a test session at the Warneford Hospital, Oxford. At the start of this session, women completed the Edinburgh Postnatal Depression Scale (EPDS) (Cox et al. 1987) and the Spielberger Trait Anxiety Inventory (Spielberger et al. 1983) and a demographic questionnaire.

Women were then shown a short film depicting distressed young infants. The film was 6 min in length and included eight consecutive short clips of infants crying, displayed on a computer screen. The infants in the film were all under 6 months of age. The clips were taken from online sources, with permission from the owners. Women viewed the film alone, and were asked to sit quietly and watch the screen.

Saliva samples were collected at five time points during the test session using salivette tubes (Sarstedt, Leicester, U.K.) Two samples were taken before the film, one approximately 30 min before the film and one immediately before the film. The third sample was taken immediately following the end of the film; the fourth and fifth samples were taken 15 and 30 min following the end of the film, respectively.

Mood ratings were taken immediately before and after the film, by asking the participants to complete the Spielberger State Anxiety Inventory (Spielberger et al. 1983) and the Positive and Negative Affect Scale (Watson et al. 1988). After the film, participants completed three visual analogue scales rating ‘how much did you want to comfort the babies?’, ‘how upsetting did you find the film?’ and ‘how good do you think you would be at comforting the baby?’.

### Biochemical analysis

Saliva samples were stored at −20 °C until analysis. Saliva cortisol analysis was carried out by a direct double-antibody radioimmunoassay (RIA) with utilisation of 125I-cortisol as the ligand. The intra- and inter-assay coefficients of variation (CVs) were 4.1 and 7.8 %, respectively, and the minimum detectable concentration was 0.2 nmol/l when a 0.1-ml volume was assayed.

### Depressive symptoms

The EPDS was used to divide participants into a ‘depressed symptom’ group and a control group. The EPDS is a self-report measure of depression. Although originally developed to screen for postnatal depression (Cox et al. 1987), it has subsequently been validated for use within the prenatal period (Murray and Cox 1990). It consists of 10 items covering the common symptoms of depression, but does not include somatic symptoms such as fatigue and change in appetite, which may be affected in the normal course of pregnancy. Each item is scored from 0 to 3, with a maximum score of 30. Although a range of cutoff scores has been used with this measure, a cutoff of 10 has been shown to yield a group at risk for depression (Adewuya et al. 2006; Adouard et al. 2005; Bergink et al. 2011; Bunevicius et al. 2009; Felice et al. 2004; Murray and Cox 1990). A recent study suggested that using a cutoff score of 10 provides a good balance between sensitivity (70–79 %), specificity (96–97 %) and positive predictive value (39–51 %) when the scale is used in the second trimester in an unselected pregnant sample (Bergink et al. 2011). Thus, in the current study, participants were classified into a “depressed symptom group” if they scored 10 or above on the EPDS (*N* = 14), while those scoring below 10 were classified as controls (*N* = 39).

### Analysis

The data was analysed using analysis of variance and independent samples *t* tests. A mean was taken of the two baseline cortisol measures, and a *t* test was performed to check that this did not differ between the two groups. Change from baseline cortisol scores were calculated for each group and time point by subtracting the average baseline cortisol level from the cortisol level at the three post-film time points.

## Results

### Demographic variables

Trait anxiety scores were significantly higher in the depressed symptom group compared with the control group [*t*(51) = 4.7, *p* < 0.001]. There was no difference in age [*t*(51) = 0.29, *p* = 0.8], days of gestation [*t*(51) = 1.6, *p* = 0.1] and desire to have children [*t*(51) = 1.1, *p* = 0.3] between the depressed symptom and control groups.

### Cortisol response to distressed infant film

**Fig. 1:**
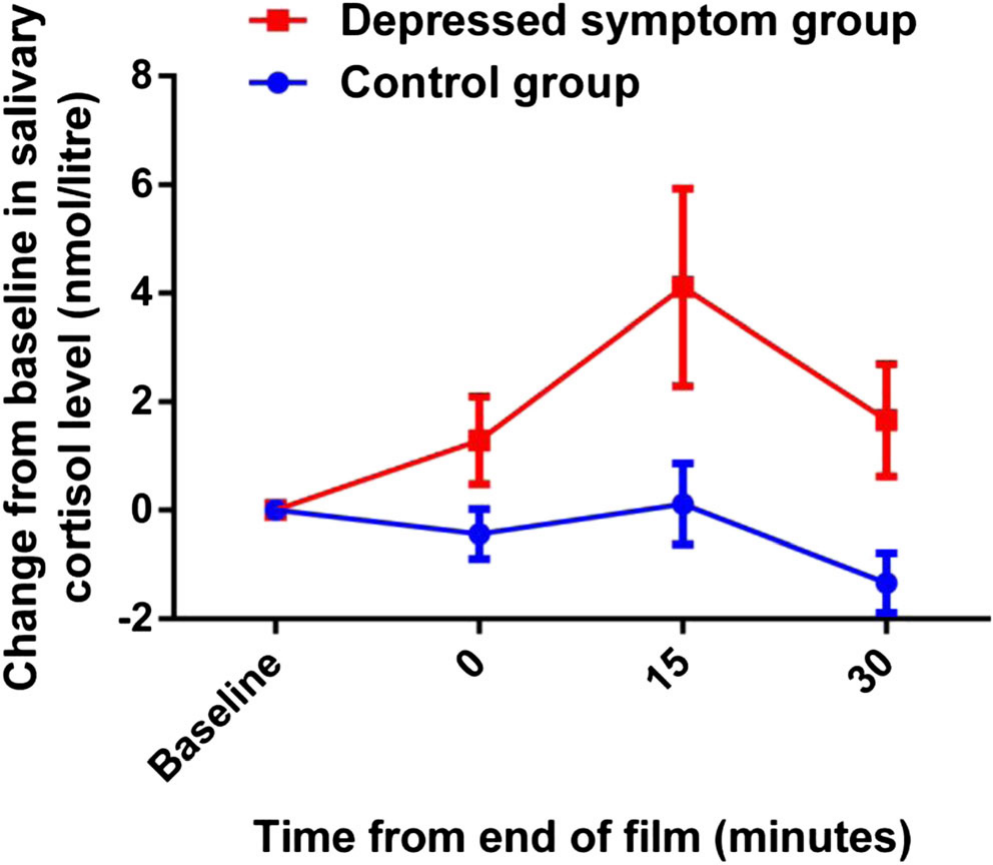
Salivary cortisol (nmol/litre) change from baseline in response to the infant distress film. *Error bars* represent SEM

Baseline cortisol concentration before the start of the film was not significantly different between the depressed symptom group (mean = 7.28 nmol/l, SD = 2.2) and the control group (mean = 6.04 nmol/l, SD = 3.3 [*t*(51) = 1.29, *p* = 0.2]). An ANOVA on scores of change from baseline showed a main effect of time [*F*(2,102) = 5.29, *p* = 0.007] and a main effect of group [*F*(1,51) = 8.23, *p* = 0.006]. The group by time interaction was not significant [*F*(2,102) = 1.53, *p* = 0.22]. The main effect of group reflected increased cortisol change from baseline in the depressed symptom group compared with the control group (see Fig. 1).

### Mood ratings in response to distressed infant film

There was a significant main effect of time showing an increase in state anxiety scores [*F*(1,51) = 42.2, *p* < 0.001] following film viewing in both groups. There was also a main effect of group [*F*(1,51) = 15.4, *p* < 0.001], which reflected relatively increased state anxiety scores in the depressed symptom group at both time points. However, there was no significant interaction between time and group [*F*(1,51) = 2.0, *p* = 0.17], suggesting that the two groups did not differ in the magnitude of change in state anxiety from pre- to post-film (see Table 2).

**Table 2:** Subjective mood response to distressed infant film

	Depressed symptom group		Control group	
	Pre-film	Post-film	Pre-film	Post-film
Spielberger state anxiety (mean, SD)	38.8 (12.9)	48.4 (12.7)	29.8 (6.5)	36 (9.6)
Positive PANAS (mean, SD)	29.9 (7.4)	28.1 (7.7)	35.2 (6.7)	35.6 (12.7)
Negative PANAS (mean, SD)	19.2 (7.3)	22.5 (8.7)	12.8 (3.9)	16.4 (14.1)
VAS “How much did you want to comfort the baby?” (mean, SD)		85.1 (23.8)		82.6 (19.1)
VAS “How upsetting did you find the film?” (mean, SD)		53.5 (24.7)		54.6 (26.1)
VAS “How good do you think you would be at comforting the baby?” (mean, SD)		65.3 (20.7)		67.3 (20.8)

*PANAS* positive and negative affect scale, *VAS* visual analogue scale

There was no significant effect of time and no significant interaction between time and group on either positive or negative scores on the Positive and Negative Affect Scale (PANAS). However, there was a significant effect of group on positive scores [*F*(1,51) = 6.66, *p* = 0.013] and negative scores [*F*(1,51) = 6.7, *p* = 0.012], reflecting decreased positive scores and increased negative scores in the depressed symptoms group at both time points compared with the control group (see Table 2).

On the visual analogue scales, there was no significant difference in the extent to which each group rated their desire to comfort the babies in the film [*t*(51) = 0.39, *p* = 0.7], how upsetting they found the film [*t*(51) = 0.14, *p* = 0.9] or how good they thought they would be at comforting the babies [*t*(51) = 0.31, *p* = 0.8].

## Discussion

The aim of the current study was to investigate whether salivary cortisol response to a distressed infant film was increased in pregnant women currently experiencing symptoms of depression compared with non-depressed controls. Although both groups showed a similar increase in self-reported state anxiety in response to the film, there was a significantly increased cortisol response in the depressed symptom group compared with the control group. This was not explained by heightened cortisol at baseline, as both groups had similar salivary cortisol levels before the film started. Such evidence is consistent with the idea that depression during pregnancy is associated with increased reactivity of the HPA axis in response to stress. This may have important implications for our understanding of the mechanisms by which prenatal maternal mood disturbance can impact upon fetal development and child postnatal outcomes.

An alteration of the HPA axis of both the mother and child has been proposed as a potential underlying biological mechanism linking prenatal maternal mood disturbance with adverse offspring outcomes. HPA axis function is known to be altered in infants who have been exposed to prenatal maternal anxiety and depression. For example, maternal mood disturbance during pregnancy has been shown to predict increased baseline and mean infant cortisol levels at 6 months (Brennan et al. 2008). There is also evidence that prenatal maternal anxiety is associated with HPA alterations that persist into later childhood, such as individual differences in awakening and afternoon cortisol levels in 10-year-old children (O’Connor et al. 2005), and a high, flattened cortisol daytime profile in 14- and 15-year-old adolescents (Van den Bergh et al. 2007). Evidence from animal studies has also demonstrated that the HPA axis is susceptible to the long-term effects of prenatal developmental experience. For example, the offspring of rats exposed to psychological stress whilst pregnant have been reported to show enhanced emotionality in the open field test, depression-like behaviour in a forced swim test, and enhanced activity of the HPA axis, compared to the offspring of unexposed dams (Ansorge et al. 2007).

This body of evidence supports the theoretical model that maternal depression and anxiety during pregnancy may result in ‘programming’ of the infant HPA axis during fetal development, such that it is overactive and predisposes offspring to adverse behavioural and emotional outcomes. Increased levels of cortisol in the mother could potentially mediate such effects of depression and anxiety on the development of the foetal HPA system. Foetal exposure to maternal cortisol is regulated by the placental enzyme 11β-hydroxysteroid dehydrogenase type 2 (11β-HSD2), which oxidises cortisol into its inactive form, cortisone (Beitins et al. 1973; Brown et al. 1996). However, whilst this enzyme provides partial protection for the fetus from maternal cortisol levels, some active cortisol continues to pass through the placenta and fetal cortisol levels are significantly correlated with maternal levels throughout pregnancy (Gitau et al. 1998, 2001). Further, high levels of stress during pregnancy are associated with a downregulation of this enzyme, so that even more cortisol crosses the placental barrier and enters the fetal blood circulation (O’Donnell et al. 2012). The HPA axis undergoes dramatic changes during pregnancy, regardless of maternal mood. This is largely due to the release of corticotrophin releasing hormone (CRH) from the placenta, which increases exponentially during pregnancy resulting in up to a 1000-fold increase in CRH at term. Thus, as pregnancy progresses, a state of hypercortisolism develops, whereby plasma concentrations of cortisol are 2–3-fold higher than in non-pregnancy by term.

Despite increases in cortisol levels during pregnancy, there is evidence that salivary cortisol responses to acute stressful stimuli seem to attenuate as pregnancy progresses (De Weerth et al. 2007; Entringer et al. 2010; Kammerer et al. 2002). For example, Kammerer et al. (2002) reported no increase in salivary cortisol in 10 pregnant women who completed the cold hand stressor test during the third trimester of pregnancy, whereas a non-pregnant control group showed a significant increase in salivary cortisol concentration in response to the same paradigm. Similarly, a larger study assessed stress reactivity in 148 pregnant women during the second and third trimester using the Trier Social Stress Test (TSST). In this population of participants, the TSST did not induce an increase in salivary cortisol at either of the time points, though a significant increase was evident in a group of 31 non-pregnant control participants (Entringer et al. 2010). Conversely, three studies have reported significant salivary cortisol responses to stressful stimuli in pregnancy, using both psychological and physical stressors (De Weerth et al. 2007; Nierop et al. 2006; Saisto et al. 2004). However, although these studies did not include a non-pregnant control group, the magnitude of the response was lower than expected for a non-pregnant population.

Such evidence of reduced HPA axis responses to stress during pregnancy (De Weerth et al. 2007; Entringer et al. 2010; Kammerer et al. 2002) is consistent with studies that report reduced cardiovascular responses to stress in pregnant women. It is possible that such decreased stress reactivity in pregnancy is an adaptive response; over exposure to maternal stress hormones and alterations in cardiovascular responses, including uterine blood flow, may disrupt normal fetal development. Thus, an increased baseline of the stress response systems may buffer fluctuations in maternal stress reactivity, and protect the fetus. Therefore, individual differences in maternal stress reactivity during pregnancy may have implications for foetal development. However, a significant gap in the literature concerns the effect of maternal mood on the responsivity of the HPA axis to stress. The current study importantly provides early evidence to suggest that, whilst HPA axis responses to stress are in general attenuated during pregnancy, there continues to be a robust salivary cortisol response to an acute stressor in women who are experiencing current symptoms of depression. Such evidence is consistent with a recent report of increased systolic blood pressure responses towards infant distress in pregnant depressed women compared with non-depressed controls (Pearson et al. 2012). Taken together, these studies raise the intriguing possibility that depression during pregnancy is associated with a failure to demonstrate the usual attenuation of physiological responses to acute stress, which may be an important factor when considering alterations in the foetal environment that may be associated with disturbed maternal prenatal mood.

The evidence presented here should be interpreted in light of a number of methodological limitations of the current study. First, the depressed symptom group was categorized according to scores on a self-report measure of depression (Edinburgh Postnatal Depression Questionnaire) rather than a clinical interview. Whilst this is a widely used and validated scale, it is important to acknowledge that it is not known whether all of the women in the depressed symptom group would have met formal diagnostic criteria for a current episode of depression. However, the clear and significant differences in salivary cortisol response to the distressed infant film between the two groups suggest that the mood disturbance in the depressed symptom group was sufficient to impact upon HPA function. Second, there was a significant difference in trait anxiety scores between the two groups, meaning that it is not possible to differentiate the effect of anxiety and depression on salivary cortisol response to the film. However, depression and anxiety are known to be highly comorbid, and future studies with a larger sample size may be able to more clearly establish whether depression and anxiety have dissociable effects on HPA axis response to stress during pregnancy or act via a common negativity pathway. Third, it remains unknown if depression-related changes in salivary cortisol response to an acute stressor persist to later stages of pregnancy. All of the women in the current study were tested between 11 and 18 weeks gestation. Since the HPA axis is known to undergo dramatic changes later in pregnancy, an important outstanding question is whether maternal depression continues to be associated with increased cortisol reactivity to stress at later time points. Further, it remains to be determined whether there is a critical time point for the fetus to be affected by increased cortisol secretion during development. Finally, whilst the current study used an infant-related stressor, it is unclear whether similar effects would also be evident in response to a stress challenge unrelated to infant distress.

In summary, the current study found that women experiencing symptoms of depression during pregnancy have heightened salivary cortisol responses to stimuli involving infant distress. This is an important demonstration of depression-related changes in HPA axis function and supports the idea that hyper-responsivity of this system to stress during pregnancy is a potential mechanism by which maternal mood disturbance during pregnancy may impact upon foetal development and later child behavioural and emotional outcomes.
